# A Multiparametric Assay Platform for Simultaneous In Vivo Assessment of Pronephric Morphology, Renal Function and Heart Rate in Larval Zebrafish

**DOI:** 10.3390/cells9051269

**Published:** 2020-05-20

**Authors:** Petrus J. Steenbergen, Jana Heigwer, Gunjan Pandey, Burkhard Tönshoff, Jochen Gehrig, Jens H. Westhoff

**Affiliations:** 1 Department of Pediatrics I, University Children’s Hospital Heidelberg, 69120 Heidelberg, Germany; petrus.steenbergen@embl.de (P.J.S.); jana.heigwer@med.uni-heidelberg.de (J.H.); gunjan.pandey@med.uni-heidelberg.de (G.P.); burkhard.toenshoff@med.uni-heidelberg.de (B.T.); 2 DITABIS, Digital Biomedical Imaging Systems AG, 75179 Pforzheim, Germany; 3 ACQUIFER Imaging GmbH, 69123 Heidelberg, Germany

**Keywords:** zebrafish, automated imaging, screening, developmental toxicity, pronephros, kidney function, kidney morphology, heart rate

## Abstract

Automated high-throughput workflows allow for chemical toxicity testing and drug discovery in zebrafish disease models. Due to its conserved structural and functional properties, the zebrafish pronephros offers a unique model to study renal development and disease at larger scale. Ideally, scoring of pronephric phenotypes includes morphological and functional assessments within the same larva. However, to efficiently upscale such assays, refinement of existing methods is required. Here, we describe the development of a multiparametric in vivo screening pipeline for parallel assessment of pronephric morphology, kidney function and heart rate within the same larva on a single imaging platform. To this end, we developed a novel 3D-printed orientation tool enabling multiple consistent orientations of larvae in agarose-filled microplates. Dorsal pronephros imaging was followed by assessing renal clearance and heart rates upon fluorescein isothiocyanate (FITC)-inulin microinjection using automated time-lapse imaging of laterally positioned larvae. The pipeline was benchmarked using a set of drugs known to induce developmental nephrotoxicity in humans and zebrafish. Drug-induced reductions in renal clearance and heart rate alterations were detected even in larvae exhibiting minor pronephric phenotypes. In conclusion, the developed workflow enables rapid and semi-automated in vivo assessment of multiple morphological and functional parameters.

## 1. Introduction

Preclinical studies on drug discovery, drug-induced developmental toxicity and safety of chemical compounds require convenient animal models in combination with accurate technologies and reliable test assays. Despite yielding the highest predictivity, preclinical toxicology testing in mammalian models is laborious, time consuming, cost intensive, and burdened with ethical questions concerning animal testing [1,2]. In contrast, the zebrafish (Danio rerio) is in line with the 3Rs (replacement, reduction, and refinement) concept in animal research [3,4], and offers a cost-effective non-mammalian vertebrate model system [5]. With its small, microtiter plate-compatible size, optical transparency, ex utero development and rapid organogenesis, zebrafish embryos and larvae have been established as a valuable model for large-scale in vivo chemical screening studies, both for toxicological and for phenotypic drug screening approaches [5,6,7]. Hence, the zebrafish model can occupy a niche between in vitro models and mammalian biomedical models [8].

The zebrafish larval pronephros, the earliest nephric stage, consists of two nephrons with a fused glomerulus at the midline [9]. Osmoregulation and blood filtration in the larval pronephros start as early as 48 h post-fertilization (hpf). Despite simpler morphology and tremendous differences in nephron number between humans and zebrafish embryos/larvae, there is homology concerning genetic, structural and functional aspects [10]. Additionally, on the single-nephron level, the zebrafish pronephros is composed of highly differentiated and segmentally organized glomerular and tubular cells that largely resemble human kidney cells with respect to genetics, metabolism, physiology and morphology, indicating evolutionary conservation [9,10,11]. Hence, the zebrafish can serve as a model system for the study of renal development, kidney disease, and nephrotoxicological studies [5,6,12,13]. Abnormal renal development can include functional impairment and morphological alterations—both of which can occur independently or at the same time. Therefore, to fully exploit the in vivo context, newly developed assay platforms for pronephric evaluation should combine scoring of both morphological and functional phenotypes.

Using the transgenic *Tg(wt1b:EGFP)* zebrafish line with pronephros-specific reporter gene expression, we have previously established a high-content and large-scale compatible in vivo screening pipeline for the morphological analysis of the larval zebrafish kidney [14,15]. For functional analysis, microinjection techniques as well as transgenic zebrafish lines allow for the assessment of glomerular filtration barrier integrity [16,17,18] and renal clearance [19,20], either alone or in a combinatorial approach [13,21]. Recently, Gorgulho et al. published a dedicated pipeline for the evaluation of drug-induced functional and morphological renal tubular alterations [22]. While renal assays in that elegant study included, among others, both investigation of renal function by the inulin clearance assay and the assessment of detailed 3D tubular morphology by two-photon microscopy, they did not allow parallel or automated in vivo assessment of both parameters within the same larva. 

To this end, we developed a novel multiparametric assay platform on an automated screening microscope that allows for the in vivo assessment of both renal clearance and pronephros morphology within the same zebrafish larva in a semi-automated fashion. In addition, we implemented a tool for heart rate determination. The established pipeline was evaluated in a pilot screen investigating a set of previously described drugs known to induce both human and zebrafish developmental nephrotoxicity [15]. Here, drug-induced renal developmental toxicity included, beyond pronephros malformation, reductions in renal clearance and heart rate alterations. In summary, the developed pipeline provides a novel implementation of a semi-automated, multiparametric in vivo renal function and morphology assay that includes heart rate measurement as an additional factor with an impact on renal clearance.

## 2. Materials and Methods

### 2.1. Ethics Statement

The work presented does not involve work with animals according to German and European legislation. All experiments were performed at stages prior to the legal onset of animal life. To obtain zebrafish embryos and larvae, fish were maintained in closed stocks at the Karlsruhe Institute of Technology (KIT). All the zebrafish husbandry and experimental procedures were performed in accordance with the German animal protection regulations (Regierungspräsidium Karlsruhe, Germany; Tierschutzgesetz 111, Abs. 1, Nr. 1, AZ35-9185.64/BH). The facility is under the supervision of the Regierungspräsidium Karlsruhe.

### 2.2. Fish Keeping and Embryo Handling

Adult zebrafish of the *Tg(wt1b:EGFP)* transgenic line [23] were maintained at 28 °C on a 14 h light/ 10 h dark cycle. Eggs were collected from pairwise and batch crossings and kept at 28 °C in E3 buffer. The developmental stage of embryos was determined as previously described [24]. At 24 hpf, GFP-positive embryos were selected using a stereomicroscope and enzymatically dechorionated using 10 mg/mL pronase (Sigma-Aldrich, Taufkirchen, Germany), washed twice with 400 mL of E3 buffer and transferred into 12-well plates (Corning, Wiesbaden, Germany) for subsequent drug treatment.

### 2.3. Drug Treatment of Embryos

The 24 hpf dechorionated embryos were exposed to drugs dissolved in E3 buffer containing 0.003% 1-phenyl-2-thiourea (PTU, Alfa Aesar, Karlsruhe, Germany) and 5 mM 4-(2-hydroxyethyl)-1-piperazineethanesulfonic acid (HEPES, Sigma-Aldrich, Taufkirchen, Germany) over a 24 h period at 28 °C in 12-well plates via submersion. Untreated embryos kept in E3/PTU (0.003%)/HEPES (5 mM) served as control. The total number of embryos that underwent drug treatment is shown in Table S1. The following drugs and concentrations were used: acetaminophen (2.5 and 5 mM) (Caesar und Loretz, Hilden, Germany), ampicillin sodium salt (20 and 40 mM) (AppliChem, Taufkirchen, Germany), gentamicin sulfate (7 and 14 mM) (Sigma-Aldrich, St. Louis, MO, USA), indomethacin sodium salt (10 and 15 µM) (AppliChem, Taufkirchen, Germany), kanamycin sulfate (20 and 40 mM) (AppliChem, Taufkirchen, Germany), losartan potassium salt (5 and 10 mM) (Molekula, Gillingham, Dorset, United Kingdom), and penicillin G potassium salt (10 and 20 mM) (AppliChem, Taufkirchen, Germany). Following drug treatment, the number of dead larvae was assessed, and living larvae were transferred to E3/PTU/HEPES. Based on pilot experiments, the highest drug concentration was set at a level where no gross malformations (e.g., severe edema, spinal curvature or somite malformations) were observed. Lower concentrations were used to test for effects on renal function and heart rate in a setting with only the mildest drug effects on renal phenotypes. For imaging and microinjection, 0.025% tricaine (MS-222, Sigma-Aldrich, Taufkirchen, Germany) was added to the medium. Tricaine exposure of larval zebrafish covered a time period from 74 to 101 hpf.

### 2.4. Generation of a 96-Well Dorsal-Lateral Template Tool

For standardized visualization of larval zebrafish pronephroi and vasculature, a 3D-printed orientation tool was designed that combines previously published designs for lateral and dorsal orientation [25]. The tool can be used to generate two grooves that are perpendicular to each other in agarose in the very same well, thus allowing for consistent dorsal and lateral positioning of larvae without additional transferring steps.

### 2.5. Preparation of Agarose Molds in Microtiter Plates

Each well of a 96-well microtiter plate (Cat.-No. 655087, Greiner, Frickenhausen, Germany) was filled with 75 µL of 1% agarose in E3 medium containing 0.025% tricaine using a multichannel pipette. The plate was kept at room temperature for one minute. The 3D-printed template was inserted to generate two perpendicular grooves for dorsal and lateral orientation. The tool was removed after agarose solidification and the plate was wrapped in wet paper towel and stored in a plastic bag at 4 °C until usage. Anesthetized embryos were transferred in 100 µL E3 with PTU and manually arrayed and oriented under a stereomicroscope.

### 2.6. Fluorescein Isothiocyanate (FITC)-Inulin Injection

Following dorsal imaging at 74 hpf, larvae were manually re-oriented to a lateral position that was optimal for microinjections. Fluorescein isothiocyanate (FITC)-inulin 5% *w/v* (FITC-inulin, F3272, Sigma-Aldrich, Taufkirchen, Germany) was prepared as described [20]. The injection solution contained 1 µL FITC-inulin, 1 µL 0.1 M KCl, 0.05% phenol red and 1 µL demineralized water. Injection into the common cardinal vein was performed in 96 larvae of a multiwell plate at lateral position at 76 hpf using glass injection needles. The same needle was used for each 96-well-plate experiment to exclude a potential bias by differences in injection volume. Injections were performed free-hand to increase injection speed. Using this method, we were able to inject 96 larvae in 30 min with an injection efficiency of on average 75% within the 96-well plate. Directly after injection, larvae were manually re-oriented if needed for optimal lateral imaging.

### 2.7. Image Acquisition

Overview images of larvae were taken using a stereomicroscope (SMZ1500, Nikon, Düsseldorf, Germany) 0 and 24 h after end of compound exposure. For automated acquisition of dorsal and lateral views, larvae were imaged on an ACQUIFER Imaging Machine (ACQUIFER Imaging GmbH, Germany), a widefield high-content screening microscope equipped with a white LED array for brightfield imaging, a LED fluorescence excitation light source, a sCMOS (2048 × 2048 pixel) camera, a stationary plate holder in combination with moving optics and a temperature-controlled incubation lid. For dorsal imaging, brightfield (20% relative LED intensity, 10 ms integration time) and GFP (100% relative LED intensity, 100 ms integration time) z-stack images (10 slices with 15 µm slice distance) were acquired using a 4× NA 0.13 objective (Nikon, Düsseldorf, Germany). For assessing renal clearance, five serial BF images (25 % relative LED intensity, 5 ms exposure time, 5 slices with 0 µm slice distance) and one GFP image (100% illumination power, 200 ms exposure time) were acquired using the 4x objective at 0, 2, 4, 14 and 24 h after FITC-inulin injection. To measure heart rate, lateral imaging of the heart region (4× objective, 30 serial BF images with 0 µm slice distance) was performed at 81 hpf. Images were acquired with 5 ms exposure time, resulting in a sampling rate of 13 frames per second [26]. In all experiments, the focal plane was detected in the BF channel using a built-in software autofocus algorithm (4× objective, 2 × 2 binning, 10% relative LED intensity, 10 ms exposure time, 10 slices with 45 µm slice distance).

### 2.8. Data Handling and Visualization

Image data was stored and processed on an ACQUIFER HIVE (ACQUIFER Imaging GmbH, Germany). Raw images of fluorescence channels were processed using custom-written Perl scripts in combination with Fiji macros (available upon request). These scripts and Fiji macros generated multilayer z-stacks, XY-cropped maximum projections for each experimental embryo and thumbnail montage images for each experimental microplate, readily allowing visual assessment and comparison of pronephric phenotypes as previously described [14,15].

### 2.9. Image Analysis

For renal phenotyping, quantitative measurements of kidney alterations were carried out for five morphological pronephric parameters: glomerular separation, glomerular height, glomerular width, tubular diameter and tubular distance. To measure these features, cropped maximum projection data of fluorescently labelled kidney was loaded in Fiji, 16 reference points were set manually on each projection [27], and the geometrical parameters (distances) were calculated automatically using a Fiji macro. Following normalization to quantitative measurements in control larvae that were co-assessed in each 96-well plate, heatmaps were generated using Graphpad Prism (Version 8.3.1, La Jolla, CA, USA). Fiji macros for measuring distances (pixels) in the cropped kidney images are available upon request. 

**Figure 1:**
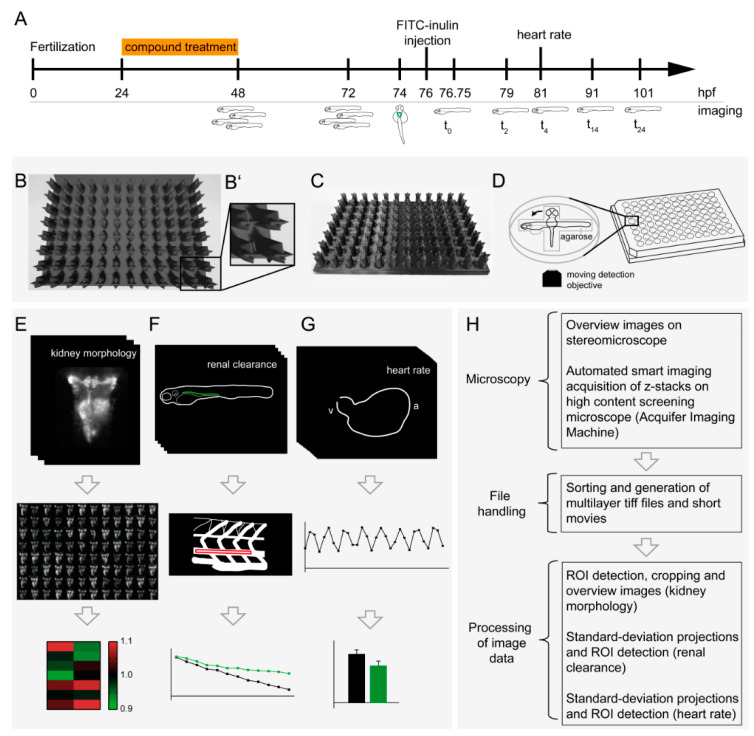
Multiparametric in vivo workflow for the assessment of renal morphology, renal clearance and heart rate. (**A**) Timeline of the workflow for drug exposure and screening of larval kidneys. Overview images were taken at 48 h post-fertilization (hpf) and at 72 hpf. Dorsal imaging of kidney morphology was carried out at 74 hpf, prior to FITC-inulin injection. Lateral fluorescent images were taken automatically after FITC-inulin injection of 96 larvae of a multiwell plate (t_0_), at 2 h post-injection (hpi, t_2_), 4 hpi (t_4_), 14 hpi (t_14_) and 24 hpi (t_24_). At t_4_, heart rate was assessed using brightfield imaging. (**B**) Renderings and (**C**) photographs of the 3D-printed orientation tool for generation of standardized grooves for lateral and dorsal positioning of zebrafish larvae within a single agarose-filled microwell: (**B**) top view and (**C**) oblique view. (**D**) Schematic representation of a dorsally orientated embryo that can easily be re-positioned within the same well to a lateral orientation. For each embryo, kidney morphology (**E**), renal clearance (**F**), and heart rate (**G**) were assessed, and analyzed data was visualized. (**H**) The flowchart depicts image processing after microscopy. Abbreviations: ROI, region of interest; a, atrium; v, ventricle.

Renal clearance was assessed following FITC-inulin injection (Figure 1F) [20]. Standard deviation z-projection was used on serial brightfield images to generate an image highlighting changing pixels caused by moving erythrocytes in the bloodstream. Using this method, the vasculature of the larva could be robustly visualized. A polygon region of interest (ROI) was manually placed over somite 16–18 in the dorsal aorta above the area of the yolk sac extension. This ROI was then restored on the image with the FITC signal and the fluorescent signal intensity (mean gray level) within this region was measured using Fiji. For background subtraction, the fluorescent signal outside the larva using an identically sized ROI placed in a fixed x and y distance outside the larva was measured. The background-corrected signal for each larva at t = 0 h after injection of a full 96-well plate was set at 100% and the signal at later time points was displayed as a percentage of the initial signal intensity. In this way, we corrected for minor variations in injection volume and injection efficiency between the individual larvae and between experiments. Thus, clearance of inulin was calculated as the percentage decrease in FITC intensity on the caudal artery using the formula (∆ actual FITC intensity/∆ FITC intensity at baseline (0 h)) × 100, whereas ∆ is the difference between vascular and background fluorescence intensity. Fiji macros are available upon request. Concentrations used for the different treatments and the numbers of analyzed embryos are listed in Table S2. Of note, few larvae that died during the imaging process and larvae that were out of focus for imaging were excluded from the analysis (Table S5).

Heart rate was evaluated by generating a movie from 30 brightfield images of larvae in lateral orientation at 4 h post-injection (hpi) of FITC-inulin solution. Intensity normalization was performed for each individual image to correct for minor intensity changes across slices. This increased the robustness of detecting changing pixels over time, as minor fluctuations in overall intensity across images caused by technical variations were eliminated. It also improved the robustness of subsequent thresholding steps. Standard deviation z-projection in Fiji was used to obtain an image highlighting changing pixels caused by moving structures allowed readily recognizing the heart region of the larvae. A small circle selection was manually placed over the heart area and restored in the original grayscale image sequence. Images were converted to 8 bit, and the total area of pixels above a threshold of 90 in each slice was measured. Total area above threshold was plotted in excel as a line graph. The pixels change followed a sinusoid movement caused by the rhythmic beating of the heart. The number of peaks in the 30 images was divided by the number of slices between the first and last peak position of the line graph and the result was converted to beats per minute. Few cases in which heart rhythm could not be robustly detected were excluded from the analysis (Table S6).

Extra-renal phenotyping upon compound treatment included the assessment of mortality and pericardial edema that were assigned based on manual evaluation of treated embryos on a stereomicroscope prior to mounting in agarose-filled microplates.

### 2.10. Statistical Analysis

For kidney morphology and heart rate assessment, normalized means among treatment groups were compared using one-way ANOVA with Dunnett correction for multiple comparisons as a post-hoc test. For renal clearance, the means among treatment groups were compared using two-way ANOVA with Tukey correction for multiple comparisons as a post-hoc test. Error bars indicate standard deviation. *p* < 0.05 was regarded as statistically significant. Data was evaluated using GraphPad Prism Version 8.3.1.

## 3. Results

### 3.1. Multiparametric In Vivo Pipeline for the Assessment of Renal Morphology, Renal Clearance and Heart Rate

We adapted and further developed our previously published protocol for automated in vivo imaging of dorsal views of zebrafish larval kidneys [15]. In brief, 24-hpf-old *Tg(wt1b:eGFP)* zebrafish embryos were exposed over a period of 24 h to a previously investigated set of FDA-approved drugs affecting kidney development in humans and zebrafish (Figure 1A) [15].

Brightfield overview images were obtained using a stereomicroscope directly after drug exposure (48 hpf) and at 72 hpf. Hereby, direct effects of compound treatment on gross morphology (i.e., development of pericardial edema) were assessed (Table S1). At 74 hpf, larvae were anaesthetized and placed in single wells of an agarose-filled 96-well plate, each well containing two grooves perpendicular to each other for specimen orientation (Figure 1B,C). This allowed for precise and consistent dorsal and lateral positioning of 3 dpf larvae into agarose molds. Due to this 2-groove-template, larvae did not need to be transferred to a different well to be imaged from varying orientations. Hence, this novel pipeline allows both morphological and functional assessment of the same larva within the same well, thereby eliminating the risk of inadvertent larval exchange during transfer and reducing the time needed. Following automated dorsal imaging (Figure 1E) [15], zebrafish larvae were repositioned to a lateral position (Figure 1D,F) for FITC-inulin injection for subsequent assessment of renal clearance. In humans, inulin clearance is the gold standard renal clearance technique for the assessment of glomerular filtration rate. Each fish was automatically imaged after injection (t_0_) and at 2, 4, 14 and 24 hpi. FITC intensity was measured within a ROI spanning somite 16–18 in the dorsal aorta above the area of the yolk sac extension (Figure 1A,F). 

Image analysis was partially automated using macros in Fiji, while ROI selection was performed manually. Heart rate was assessed using Fiji by analysis of changing pixel intensities in the heart region in serial brightfield images at 5 hpi of FITC-inulin. Graphical visualization of plotted values in a time-dependent manner allowed for determination of heart rate (Figure 1G). The general setup for the multiparametric imaging pipeline is shown in Figure 1H.

### 3.2. Impact of Compound Treatment on Pronephros Development

**Figure 2:**
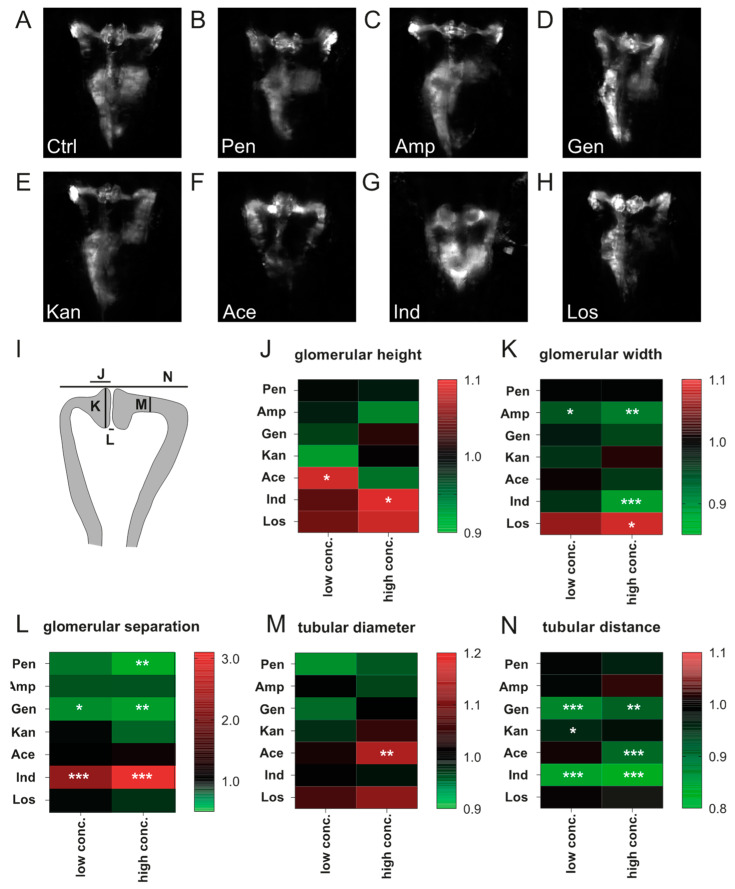
Impact of compound exposure on kidney morphology. Representative images of 3 dpf zebrafish pronephroi: (**A**) control, (**B**) 20 mM penicillin, (**C**) 40 mM ampicillin, (**D**) 14 mM gentamicin, (**E**) 40 mM kanamycin, (**F**) 5 mM acetaminophen (**G**) 15 µM indomethacin, and (**H**) 10 mM losartan. (**I**) Scheme showing respective areas of semi-automated measurements given in J–N. (J–N) Heatmaps showing log^2^ normalized data for (**J**) glomerular height, (**K**) glomerular width, (**L**) glomerular separation, (**M**) tubular diameter, and (**N**) tubular distance. Drug concentrations used and the number of larvae assessed are given in Table S2. Color codes indicate a relative increase (red) or decrease (green) in comparison to control measurements (black, value 1.0). Values are expressed as the mean. *p < 0.05, **p < 0.01, ***p < 0.001 versus non-treated control larvae by one-way ANOVA with post-hoc Dunnett test for multiple comparisons. Abbreviations: Ace, acetaminophen; Amp, ampicillin; Ctrl, control; Gen, gentamicin; Ind, indomethacin; Kan, kanamycin; Los, losartan; Pen, penicillin.

While morphological analysis was performed by a combination of quantitative and qualitative parameters that each had to be determined separately in a time-consuming manner in our previous work [15], in the present project, quantitative measurements of renal parameters were carried out by loading fluorescent image data into Fiji and manually setting 16 reference points in the images for automated calculation of the geometrical parameters using a Fiji macro [27]. Representative images of pronephroi of 75-hpf-old drug-exposed larvae are shown in Figure 1E and Figure 2.

Exposure to drug concentrations listed in Table 1 provoked only minor effects on pronephric development for penicillin G or ampicillin sodium salt. Compared to control larvae, glomerular distance was reduced at 20 mM penicillin G administration (*p* < 0.05) (Figure 2A,B,L). Glomerular width was decreased following ampicillin sodium salt exposure at 20 mM (*p* < 0.05) and 40 mM (*p* < 0.01) (Figure 2C,K). Following gentamicin sulfate administration, glomerular separation was more pronounced (*p* < 0.01 for 7 mM and 14 mM) and tubular distance decreased (*p* < 0.001 for 7 mM, *p* < 0.01 for 14 mM) (Figure 2D,L,N). Kanamycin at 20 mM slightly reduced tubular distance in comparison to control larvae (*p* < 0.05) (Figure 2E,L). Acetaminophen increased glomerular height (*p* < 0.05 for 2.5 mM), decreased tubular distance (*p* < 0.0001 for 5 mM) and increased tubular diameter (*p* < 0.01 mM for 5 mM) (Figure 2F,J,L,M). Indomethacin increased glomerular height (*p* < 0.05 for 15 μM), decreased glomerular width (*p* < 0.001 for 15 μM), increased glomerular distance (*p* < 0.001 for 10 μM and 15 μM) and reduced tubular distance (*p* < 0.001 for 10 μM and 15 μM) (Figure 2G,J,K,L,N). Losartan at concentration of 10 mM increased glomerular width compared to untreated larvae (*p* < 0.05) (Figure 2H,K). The total number of analyzed zebrafish is listed in Table S2. Quantitative data of the morphological parameters is also presented in Tables S3 and S4.

### 3.3. Impact of Tested Compounds on Pronephric Clearance

**Figure 3:**
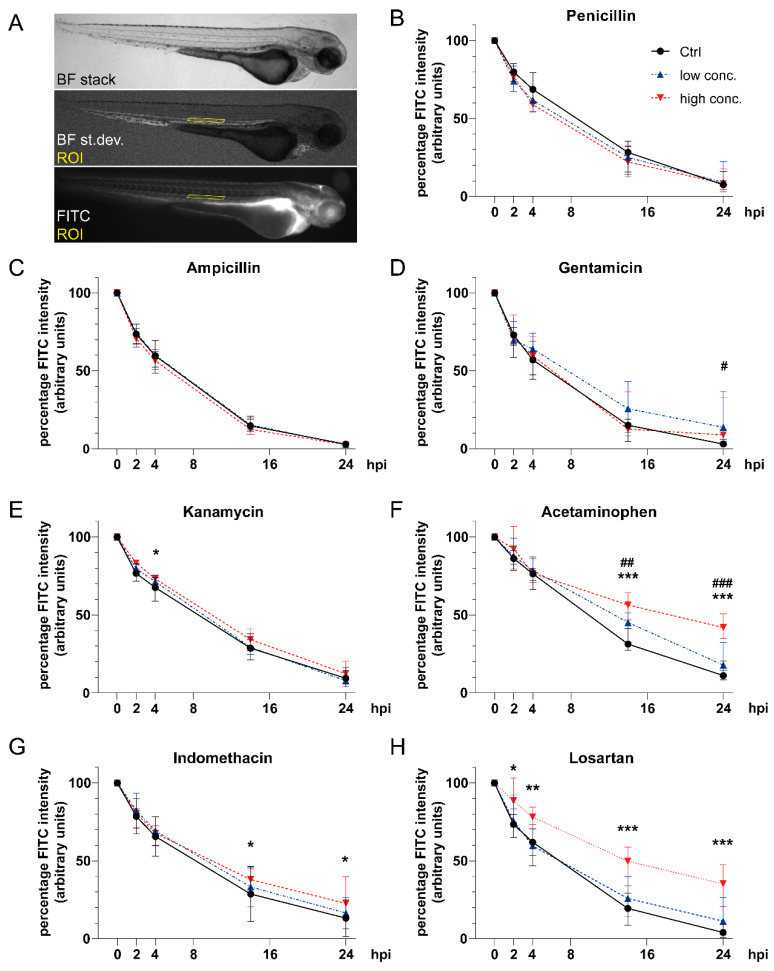
Impact of compound exposure on renal fluorescein isothiocyanate (FITC)-inulin clearance. (**A**) Lateral images of an 81-hpf-old zebrafish larva to illustrate image acquisition and definition of ROI. (**B**–**H**) Line graphs showing decrease in FITC-inulin fluorescent signal as automatically detected in the dorsal aorta at 0, 2, 4, 14 and 24 hpi for (**B**) penicillin, (**C**) ampicillin, (**D**) gentamicin, (**E**) kanamycin, (**F**) acetaminophen, (**G**) indomethacin, and (**H**) losartan. Line description as in Figure 3B. Numbers of analyzed embryos at different time points are given in Table S5. Values are expressed as the median and interquartile range. ^#^*p* < 0.05 low concentration (conc.) versus control (Ctrl), ^##^*p* < 0.01 low conc. versus Ctrl, ^###^*p* < 0.001 low conc. versus Ctrl, *p < 0.05 high conc. versus Ctrl, **p < 0.01 high conc. versus Ctrl, and ***p < 0.001 high conc. versus Ctrl by two-way ANOVA with post-hoc Tukey test for multiple comparisons. Abbreviations: BF, bright field; Ctrl, control; FITC, fluorescein isothiocyanate; ROI, region of interest; st.dev., standard deviation.

To be compatible with microinjection, only compound concentrations that did not result in gross overall malformations were used in this study. The percentage reduction in vascular FITC-inulin fluorescence intensity over time was measured following microinjection as a marker of renal clearance (Figure 3A) [19,20,22]. Both penicillin G and ampicillin sodium salt did not affect renal clearance (Figure 3B,C). By contrast, gentamicin exposure (Figure 3D) at a concentration of 7 mM reduced vascular FITC-inulin clearance at 24 hpi (4.4% (0 mM) versus 13.6% (7 mM), *p* < 0.05). Kanamycin at a concentration of 40 mM provoked a slight reduction in FITC-inulin clearance at 4 hpi compared to unexposed larvae (Figure 3E) of 66.1% (Ctrl) versus 72.7%, *p* < 0.05). In acetaminophen-treated zebrafish larvae (Figure 3F), a significant dose-dependent reduction in FITC-inulin clearance was detected (14 hpi: 33.3% (Ctrl) versus 42.3% (2.5 mM, *p* < 0.01) versus 55.8% (5 mM, *p* < 0.0001); 24 hpi: 13.7% (Ctrl) versus 22.0% (2.5 mM, *p* < 0.01) versus 42.5% (5 mM, *p* < 0.0001)). Indomethacin administration provoked dose-dependent reductions in FITC-inulin clearance, yielding significantly higher fluorescence intensities at a concentration of 15 μM at 14 hpi (39.5% versus 28.71% (Ctrl), *p* < 0.05) and 24 hpi (27.0% versus 13.3% (Ctrl), *p* = 0.013) compared to unexposed larvae (Figure 3G). When larvae were exposed to losartan (Figure 3H), a slower decrease in FITC-inulin fluorescence intensity was observed in a dose-dependent manner that became significant for a concentration of 10 mM at 2 hpi (91.5% versus 74.5% (Ctrl), *p* < 0.05), 4 hpi (77.5% versus 60.9% (Ctrl), *p* < 0.01), 14 hpi (47.1% versus 20.5% (Ctrl), *p* < 0.0001) and 24 hpi (34.7% versus 5.9% (Ctrl), *p* < 0.01). 

### 3.4. Impact of Tested Compounds on Larval Heart Rate

**Figure 4:**
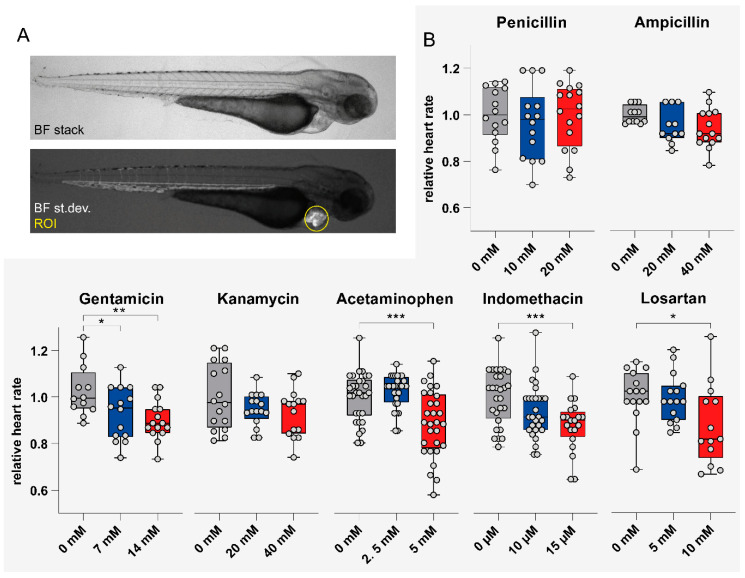
Impact of compound exposure on heart rate. (**A**) Lateral images of an 81-hpf-old zebrafish larva to illustrate image acquisition and definition of the region of interest (ROI). (**B**) Relative heart rate of zebrafish larvae following drug treatment measured at 81 hpf (i.e., 4 hpi). Values are expressed as the mean ± SD. *p < 0.05, **p < 0.01, ***p < 0.001 versus control; by one-way ANOVA with post-hoc Dunnett test for multiple comparisons. Abbreviations: BF, bright field, st.dev., standard deviation; ROI, region of interest.

Heart rate was assessed at 4 h post-FITC-inulin injection. Serial brightfield images of the cardiac region were taken automatically in laterally positioned larvae (Figure 4A). Data was analyzed as described under Methods. Video analysis revealed that heart rate normalized to unexposed zebrafish larvae was significantly reduced following acetaminophen (*p* < 0.001 for 5 mM), indomethacin (*p* = 0.001 for 15 μM), gentamicin (*p* < 0.05 for 7 mM, *p* < 0.01 for 14 mM), and losartan (*p* < 0.01 for 10 mM) exposure (Figure 4B). Penicillin G, ampicillin sodium salt and kanamycin at the given concentrations showed no significant effect on heart rate. Absolute values for heart rate assessment are shown in Table S6. For correlation of heart rate with morphological and functional parameters, see Figure S1.

## 4. Discussion

The zebrafish in biomedical research has the potential to bridge the gap between in vitro models and higher mammalian organisms in a fast and cost-saving manner [28,29]. Zebrafish screening platforms are considered alternatives to animal models in line with the 3R principles toward animal research [3,4,15]. However, these convenient animal models require large-scale screening pipelines and novel assay platforms, tailored screening protocols, easy-to-use automated imaging techniques and time-saving sample positioning tools that, due to the complex three-dimensional larval shape, enable precise automated imaging of the region of interest. Multiparametric organ-specific screening pipelines for chemical compound screening ideally enable both morphological and functional organ-specific assessments within the same organism and on a single imaging platform [28]. Hence, in this project, we developed a multiparametric, semi-automated in vivo imaging pipeline for simultaneous assessment of renal morphology, renal function and heart rate in larval zebrafish on a widefield high-content screening microscope. For this purpose, based on previous work [14,15,25], a customized 3D-printed positioning tool was further developed that generated two perpendicular grooves in agarose-filled 96-well plates allowing for consistent dorsal and lateral positioning of zebrafish embryos and larvae for subsequent renal and vascular imaging. Hereby, the risk of mixing up larvae during transfer was avoided and time for multiparametric assessment was reduced. The use of a widefield screening microscope equipped with a stationary sample holder and moving optics avoided any movement-related unintentional change of specimen orientation during automated image acquisition. By taking advantage of our easy-to-use setup, we were able to microinject FITC-inulin into 96 larvae in a microwell plate in 30 minutes with an injection efficiency of 75% on average within the 96-well plate. By comparison, e.g., Cosentino et al. injected only 15–20 larvae per hour [30]. 

In fact, inulin clearance is the gold standard for the measurement of glomerular filtration rate in humans, as it is freely filtered by the glomerulus and is neither secreted nor reabsorbed in the tubules. In zebrafish larvae, microinjection of FITC-inulin and quantification of loss of fluorescent signal from the vasculature has been previously described for the assessment of renal clearance [19,20,22]. Rider et al. injecting FITC-inulin at 100 hpf showed that vascular fluorescence correlated indirectly with FITC intensity in the surrounding medium [20].

As renal function depends on cardiovascular function, we aimed to include heart rate as an additional parameter to our multiparametric pipeline. A variety of techniques for the assessment and analysis of larval heart rate have been described [26,31,32,33,34,35,36,37,38]. In our study, we used a self-written macro in Fiji that allowed for automated measurement of changes in pixel density in the larval heart. In our semi-automated workflow, in contrast to others [20,22], brightfield images for heart rate measurement were taken expeditiously in parallel to the time-lapse renal clearance imaging workflow, and both renal and cardiac function were assessed within the same larva. 

Here, the multiparametric imaging pipeline was validated in a pilot study using a set of previously tested drugs that partly possess developmental nephrotoxicity in humans which paralleled findings in zebrafish larvae [15]. Drug concentrations for these experiments were set at a maximum level that provoked only minor morphological phenotypes, i.e., pericardial edema. We confirmed that penicillin antibiotics caused only minor effects on larval pronephric phenotypes [15]. In addition, clearance of FITC-inulin was not significantly affected by penicillin G and ampicillin sodium salt exposure via the fish water. At the same time, heart rates in both penicillin G- and ampicillin sodium salt-treated larvae remained unaltered. Aminoglycoside antibiotics including gentamicin and kanamycin have been shown to cause glomerular and tubular damage in humans, rodents and following microinjection in zebrafish [39,40,41]. In our previous study, gentamicin sulfate exposure via the fish water caused glomerular malformation and incomplete glomerular fusion at high drug concentrations. In the present screen, gentamicin sulfate caused reduced glomerular separation and tubular distance, while kanamycin, as shown before, had no significant effect on pronephros development. Reduced FITC-inulin clearances observed at 24 hpi in 7 mM gentamicin sulfate-exposed zebrafish larvae, however, demonstrated significant effects on kidney function, although transdermal uptake of aminoglycosides is considered to be low. Heart rate was significantly reduced following gentamicin exposure. While intake of acetaminophen at therapeutic doses during pregnancy and treatment of preterm newborns is largely considered safe [42], there is increasing data on developmental nephrotoxicity, in addition to hepatotoxicity, from rodent and zebrafish studies [22,43,44]. Even at the rather low drug concentrations we used for this study, renal clearance was significantly reduced in zebrafish larvae, and heart rate declined. The AT1-receptor blocker losartan was previously shown to induce abnormal pronephric development in zebrafish larvae [15]. Here, we confirmed abnormal renal development and demonstrated that renal clearance was dose-dependently reduced following losartan administration. In rats that were exposed to losartan during lactation, renal clearance has been shown to be diminished [45,46]. In addition, in our study, heart rate was significantly reduced upon losartan treatment. Indomethacin can affect kidney development in rat [39,47], in zebrafish [15,48], in an ex vivo murine embryogenic kidney model [49] and in humans [50,51,52]. In summary, the data from this multiparametric screening pipeline reinvestigating a subset of drugs used in a previous study confirms developmental nephrotoxicity of aminoglycosides, acetaminophen, losartan and indomethacin also on a functional basis. Different morphological phenotypes induced by different drugs may argue for segment-specific vulnerability and damage of the pronephros. Moreover, contrasting concentration-dependent effects may potentially be attributed to effects of hormesis. Developmental cardiotoxicity, to our knowledge, has not been described for gentamicin, acetaminophen, losartan or indomethacin, neither in humans nor in zebrafish. Hence, reductions in heart rate might reflect a secondary effect due to renal functional impairment and associated fluid overload. Additionally, no significant correlation of heart rate and renal FITC-inulin clearance could be found for the vast majority of drugs.

There are rather laborious workflow steps that could be further improved in future optimizations of the protocol, e.g., larval orientation, microinjection, identification of the region of interest for semi-automated functional measurement as well as manual setting of reference points for quantitative parameter determination in maximum projections. However, we aimed to reduce manual steps to a minimum, while keeping it easy to use and compatible with different samples. Nonetheless, simultaneous in vivo monitoring of different parameters such as renal clearance and heart rate as well as different positioning options within the same well of a microtiter plate still relevantly reduced workload and time investment. Noteworthy, by interfacing feature detection algorithms with automated microscopy, we have meanwhile established smart imaging workflows for detection, centering and zooming in on regions of interests which could also be applied to the demonstrated pipeline [14]. Second, we focused on heart rate as the primary cardiac parameter. Future workflows will ideally deal with other hemodynamic parameters including, for example, erythrocyte flow and shortening fraction, hence allowing for a more precise determination of cardiac output. Third, drug exposure via the fish water imposes another limitation of the established pipeline, as transdermal penetration can be hampered by physico-chemical properties of the chemical compound.

In conclusion, the developed pipeline provides a novel and structured example of a semi-automated, multiparametric in vivo workflow for the simultaneous assessment of kidney morphology, renal clearance and heart rate within the same larva and on a single high-content/high-throughput microscope. In a pilot study, we were able to show that in zebrafish larvae exposed to a set of previously used nephrotoxic drugs, developmental nephrotoxicity comprised alterations on a morphological and/or functional basis, partly accompanied by heart rate alterations. Of note, renal functional impairment was detected even without relevant kidney damage, and vice versa. In the end, further advances in high-throughput and high-content technology may increase the scale, reproducibility, and output of large-scale compound screens addressing kidney function.

## Figures and Tables

**Table 1:** Drug concentration used and number of embryos initially treated.

	Low Conc.	High Conc.	Embryos Treated per Group
Acetaminophen	2.5 mM	5 mM	50
Ampicillin	20 mM	40 mM	30
Gentamicin	7 mM	14 mM	30
Indomethacin	10 µM	15 µM	50
Kanamycin	20 mM	40 mM	30
Losartan	5 mM	10 mM	30
Penicillin	10 mM	20 mM	30

## Supplementary Materials

The following are available online at https://www.mdpi.com/2073-4409/9/5/1269/s1, Table S1: Gross morphology analysis in zebrafish larvae following drug treatment, Table S2: Number of larval zebrafish analyzed for kidney morphology, Table S3: Pronephric morphological parameters normalized to control, Table S4: Absolute values of pronephric morphological parameters, Table S5: Number of larval zebrafish analyzed for FITC-inulin clearance at different timepoints (t_0_-t_24_), Table S6: Number of larval zebrafish analyzed for heart rate following drug treatment, and Figure S1: Correlation graphs comparing heart rate, renal clearance and pronephric morphology following exposure to penicillin.
