# Supplementary material for: A Multiparametric Assay Platform for Simultaneous In Vivo Assessment of Pronephric Morphology, Renal Function and Heart Rate in Larval Zebrafish
Source: Cells. 2020 May 20;9(5):1269. doi: 10.3390/cells9051269 (PMC7290829; doi:10.3390/cells9051269)
Supplement: Supplementary file 1 [file cells-09-01269-s001.pdf]

Supplementary Table S1. Gross morphology analysis in zebrafish larvae following drug treatment.

				**Lethality**			**Pericardial edema**		
	**low conc.**	**high conc.**	**Embryos treated per group**	**0 mM**	**low conc.**	**high conc.**	**0 mM**	**low conc.**	**high conc.**
Penicillin	10 mM	20 mM	30	0 (0%)	0 (0%)	1 (3%)	1 (3%)	1 (3%)	0 (0%)
Ampicillin	20 mM	40 mM	30	0 (0%)	0 (0%)	0 (0%)	0 (0%)	0 (0%)	0 (0%)
Gentamicin	7 mM	14 mM	30	0 (0%)	3 (10%)	6 (20%)	2 (7%)	1 (3%)	0 (0%)
Kanamycin	20 mM	40 mM	30	0 (0%)	0 (0%)	1 (3%)	1 (3%)	0 (0%)	0 (0%)
Acetaminophen	2.5 mM	5 mM	50	0 (0%)	1 (2%)	0 (0%)	0 (0%)	0 (0%)	0 (0%)
Indomethacin	10 μM	15 μM	50	1 (2%)	0 (0%)	0 (0%)	0 (0%)	0 (0%)	4 (8%)
Losartan	5 mM	10 mM	30	0 (0%)	2 (7%)	10 (33%)	1 (3%)	0 (0%)	1 (3%)

Abbreviation: conc., concentration.

Supplementary Table S2. Number of larval zebrafish analyzed for kidney morphology.

	Glomerular height			Glomerular width			Glomerular separation			Tubular distance			Tubular diameter		
	0 mM	low conc.	high conc.	0 mM	low conc.	high conc.	0 mM	low conc.	high conc.	0 mM	low conc.	high conc.	0 mM	low conc.	high conc.
Penicillin	16	16	16	16	16	16	16	16	16	16	16	16	16	16	16
Ampicillin	15	16	16	15	16	16	15	16	16	15	16	16	15	16	16
Gentamicin	16	15	16	16	15	15	16	15	15	16	15	16	16	15	16
Kanamycin	16	16	15	16	16	15	16	16	14	16	16	15	16	16	15
Acetaminophen	30	27	30	30	27	30	30	27	30	30	27	30	30	27	30
Indomethacin	31	31	31	31	31	31	31	31	31	31	31	31	31	31	31
Losartan	16	14	14	16	14	14	16	14	14	16	14	14	16	13	15

For low and high concentrations see Table S1. Abbreviation: conc., concentration.

Supplementary Table S3. Pronephric morphological parameters normalized to control.

			Glomerular height			Glomerular width			Glomerular separation			Tubular distance			Tubular diameter		
	low conc.	high conc.	Ctrl	low conc.	high conc.	Ctrl	low conc.	high conc.	Ctrl	low conc.	high conc.	Ctrl	low conc.	high conc.	Ctrl	low conc.	high conc.
			1.0	0.992	0.984	1.0	0.993	0.993	1.0	0.715	0.592**	1.0	0.989	0.963	1.0	0.930	0.957
Pen	10 mM	20 mM	±0.13	±0.11	±0.10	±0.12	±0.11	±0.10	±0.40	±0.38	±0.09	±0.09	±0.06	±0.05	±0.18	±0.19	±0.11
			1.0	0.983	0.936	1.0	0.936*	0.909**	1.0	0.797	0.793	1.0	0.986	1.023	1.0	0.997	0.966
Amp	20 mM	40 mM	±0.12	±0.11	±0.11	±0.07	±0.09	±0.10	±0.38	±0.29	±0.37	±0.05	±0.04	±0.05	±0.15	±0.15	±0.13
			1.0	0.968	1.020	1.0	0.977	0.947	1.0	0.661**	0.621**	1.0	0.87***	0.906**	1.0	0.949	0.999
Gen	7 mM	14 mM	±0.15	±0.21	±0.19	±0.10	±0.14	±0.09	±0.40	±0.24	±0.23	±0.05	±0.08	±0.08	±0.13	±0.15	±0.15
			1.0	0.925	1.005	1.0	0.960	1.017	1.0	0.965	0.813	1.0	0.957*	0.978	1.0	0.976	1.046
Kan	20 mM	40 mM	±0.11	±0.07	±0.09	±0.11	±0.09	±0.07	±0.34	±0.71	±0.33	±0.05	±0.05	±0.04	±0.21	±0.15	±0.14
			1.0	1.087*	0.945	1.0	1.014	0.956	1.0	1.014	1.205	1.0	1.010	0.898***	1.0	1.024	1.144**
Ace	2.5 mM	5 mM	±0.12	±0.14	±0.13	±0.07	±0.10	±0.12	±0.40	±0.51	±0.65	±0.08	±0.05	±0.06	±0.15	±0.18	±0.17
			1.0	1.039	1.093*	1.0	0.962	0.886***	1.0	2.287***	3.059***	1.0	0.845***	0.823***	1.0	1.008	0.988
Ind	10 μM	15 μM	±0.14	±0.18	±0.14	±0.09	±0.13	±0.15	±0.48	±1.29	±1.20	±0.06	±0.06	±0.07	±0.14	±0.17	±0.16
			1.0	1.048	1.085	1.0	1.062	1.087*	1.0	0.963	0.874	1.0	0.998	1.010	1.0	1.061	1.116
Los	5 mM	10 mM	±0.11	±0.13	±0.14	±0.06	±0.13	±0.09	±0.38	±0.42	±0.33	±0.04	±0.06	±0.05	±0.17	±0.21	±0.21

Data is shown as mean ± standard deviation. *p<0.05, **p<0.01, ***p<0.001 *vs.* control larvae by one-way ANOVA with Dunnett correction for multiple comparisons as post-hoc test. Abbreviations: conc., concentration; Ctrl, control; Ace, acetaminophen; Amp, ampicillin; Gen, gentamicin; Ind, indomethacin; Kan, kanamycin; Los, losartan; Pen, penicillin.

Supplementary Table S4. Absolute values of pronephric morphological parameters.

			Glomerular height			Glomerular width			Glomerular separation			Tubular distance			Tubular diameter		
	low conc.	high conc.	Ctrl	low conc.	high conc.	Ctrl	low conc.	high conc.	Ctrl	low conc.	high conc.	Ctrl	low conc.	high conc.	Ctrl	low conc.	high conc.
			21.18	21.00	20.884	31.67	31.43	31.44	8.83	6.31	5.23**	139.81	138.28	134.68	6.88	6.40	6.58
Pen	10 mM	20 mM	±2.71	±2.22	±2.07	±3.84	±3.34	±2.93	±3.52	±3.32	±3.13	±13.14	±8.18	±7.50	±1.26	±1.32	±0.79
			21.00	20.65	19.66	33.09	30.98*	30.10**	5.84	4.65	4.63	137.04	135.14	140.17	7.74	7.72	7.48
Amp	20 mM	40 mM	±2.54	±2.41	±2.27	±2.37	±2.96	±3.21	±2.20	±1.72	±2.18	±7.42	±6.16	±6.60	±1.18	±1.18	±0.99
			22.15	21.44	22.59	34.95	34.16	32.75	5.13	3.39**	3.11**	143.66	125.25***	130.11**	7.76	7.36	7.75
Gen	7 mM	14 mM	±3.21	±4.62	±4.22	±3.35	±4.91	±3.28	±2.06	±1.22	±1.16	±7.12	±11.89	±11.98	±0.97	±1.13	±1.18
			20.43	18.89	20.53	30.72	29.50	31.24	4.92	4.75	4.00	141.68	135.62*	138.61	7.27	7.10	7.61
Kan	20 mM	40 mM	±2.28	±1.45	±1.92	±3.50	±2.74	±2.04	±1.69	±3.48	±1.61	±7.73	±6.89	±6.02	±1.52	±1.12	±1.05
			20.07	21.82*	18.97	33.60	33.91	32.13	5.95	5.92	7.17	141.67	143.27	127.21***	8.78	8.99	10.04**
Ace	2.5 mM	5 mM	±2.32	±2.79	±2.65	±2.37	±3.35	±3.92	±2.39	±3.06	±3.86	±11.26	±7.13	±8.42	±1.36	±1.58	±1.49
			21.33	22.15	23.30*	33.59	32.30	29.76***	5.88	13.45***	17.98***	138.28	116.81***	113.81***	7.85	7.91	7.75
Ind	10 μM	15 μM	±3.06	±3.88	±3.01	±3.08	±4.21	±4.90	±2.83	±7.55	±7.07	±7.64	±8.17	±9.24	±1.11	±1.33	±1.25
			20.56	21.55	22.30	31.73	33.71	34.49*	7.38	7.11	6.45	141.26	140.92	142.65	7.19	7.63	8.02
Los	5 mM	10 mM	±2.19	±2.16	±2.87	±1.95	±4.01	±2.87	±2.78	±3.10	±2.46	±5.89	±7.89	±7.07	±1.25	±1.51	±1.47

Data is shown as pixel mean ± standard deviation, with 1 pixel = 1.625 μm. *$p<0.05$, **$p<0.01$, ***$p<0.001$ *vs.* control larvae by one-way ANOVA with Dunnett correction for multiple comparisons as post-hoc test. Abbreviations: conc., concentration; Ctrl, control; Ace, acetaminophen; Amp, ampicillin; Gen, gentamicin; Ind, indomethacin; Kan, kanamycin; Los, losartan; Pen, penicillin.

Supplementary Table S5. Number of larval zebrafish analyzed for FITC-inulin clearance at different timepoints (t_0-t_{24}).

	**FITC-inulin injected larvae**			**t_0**			**t_2**			**t_4**			**t_{14}**			**t_{24}**		
	0 mM	low conc.	high conc.	0 mM	low conc.	high conc.	0 mM	low conc.	high conc.	0 mM	low conc.	high conc.	0 mM	low conc.	high conc.	0 mM	low conc.	high conc.
Penicillin	16	16	16	16	14	16	15	14	16	16	14	16	15	14	16	14	14	16
Ampicillin	16	16	16	14	14	15	14	14	15	14	14	15	14	14	15	13	13	15
Gentamicin	16	16	16	13	13	13	13	13	12	13	13	13	13	13	13	10	12	11
Kanamycin	16	16	16	15	15	14	15	15	14	15	15	14	15	15	14	15	15	14
Acetaminophen	32	32	32	27	27	26	27	26	22	27	26	26	27	26	25	27	26	22
Indomethacin	32	32	32	30	31	21	29	29	18	30	31	21	30	31	18	25	26	15
Losartan	16	16	16	16	14	13	16	14	13	16	14	13	16	13	13	14	12	8

For low and high concentrations see Table S1. Timepoint (t) numbers indicate hours following FITC-inulin injection. Abbreviation: conc., concentration.

Supplementary Table S6. Number of larval zebrafish analyzed for heart rate following drug treatment.

	**HR analyzed larvae**			**HR absolute values (in bpm) ± SD**		
	0 mM	low conc.	high conc.	0 mM	low conc.	high conc.
Penicillin	14	14	16	166.4 ± 19.69	160.7 ± 26.25	165.9 ± 23.25
Ampicillin	12	11	15	187.9 ± 7.17	187.6 ± 14.02	176.7 ± 16.08
Gentamicin	13	13	12	195.3 ± 20.96	177.0 ± 21.87	171.7 ± 17.64
Kanamycin	15	15	13	163.8 ± 23.04	154.9 ± 11.50	152.6 ± 16.77
Acetaminophen	27	27	25	158.0 ± 16.48	161.9 ± 11.40	140.2 ± 22.55
Indomethacin	28	28	19	147.9 ± 17.47	138.9 ± 17.05	129.7 ± 16.02
Losartan	15	14	10	150.2 ± 17.86	149.2 ± 15.74	132.0 ± 26.07

For low and high concentrations see Table S1. Abbreviations: HR, heart rate; bpm, beats per minute; SD, standard deviation; conc., concentration.

Supplementary Figure S1

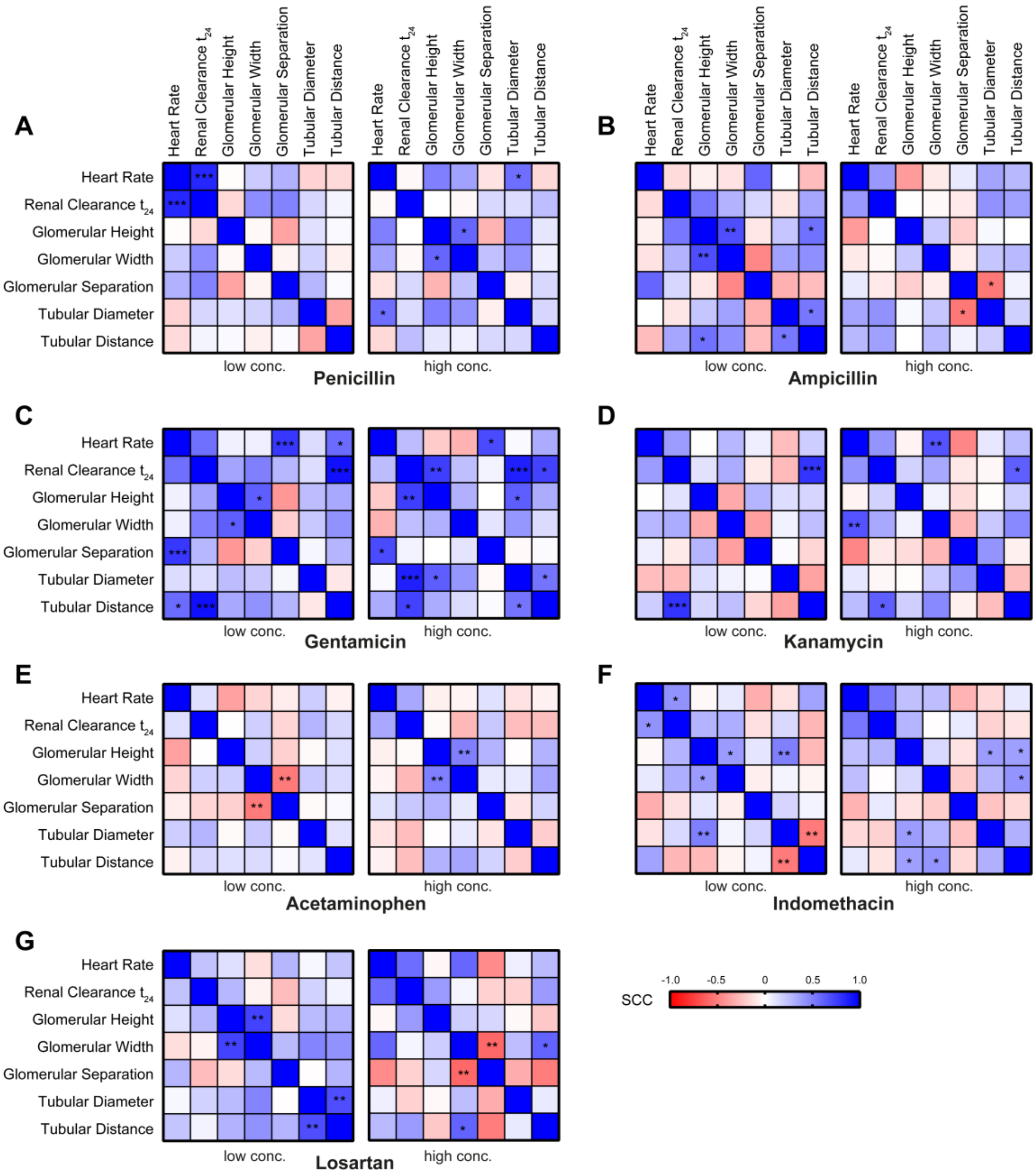

Figure S1. Correlation matrix comparing heart rate, renal clearance and pronephric morphology following exposure to penicillin (A), ampicillin (B), gentamicin (C), kanamycin (D), acetaminophen (E), indomethacin (F) and losartan (G) for low and high concentration as listed in Table 1. Color codes indicate a positive (blue) or a negative (red) Spearman's correlation coefficient (SCC). *$p<0.05$, **$p<0.01$, ***$p<0.001$. Abbreviation: conc., concentration.
